# Current State of Dry Needling Practices: A Comprehensive Analysis on Use, Training, and Safety

**DOI:** 10.3390/medicina60111869

**Published:** 2024-11-14

**Authors:** Juan Antonio Valera-Calero, Gustavo Plaza-Manzano, Gabriel Rabanal-Rodríguez, María José Díaz-Arribas, Mateusz D. Kobylarz, Jorge Buffet-García, César Fernández-de-las-Peñas, Marcos José Navarro-Santana

**Affiliations:** 1 Department of Radiology, Rehabilitation and Physiotherapy, Faculty of Nursery, Physiotherapy and Podiatry, Complutense University of Madrid, 28040 Madrid, Spain; juavaler@ucm.es (J.A.V.-C.); gusplaza@ucm.es (G.P.-M.); grabanal@ucm.es (G.R.-R.); mjdiazar@med.ucm.es (M.J.D.-A.); marconav@ucm.es (M.J.N.-S.); 2 Grupo InPhysio, Instituto de Investigación Sanitaria del Hospital Clínico San Carlos (IdISSC), 28040 Madrid, Spain; 3 Escuela Internacional de Doctorado, Universidad Rey Juan Carlos, 28922 Alcorcón, Spain; md.kobylarz@alumnos.urjc.es; 4 Akademia Terapii Manualnej i Igłoterapii Suchej (ATMIS), 34-400 Nowy Targ, Poland; 5 Faculty of Health Sciences, Universidad Francisco de Vitoria, 28223 Madrid, Spain; 6 Department of Physical Therapy, Occupational Therapy, Rehabilitation and Physical Medicine, Universidad Rey Juan Carlos, 28922 Alcorcón, Spain

**Keywords:** adverse effects, dry needling, myofascial trigger points, risk management, safety management

## Abstract

*Background and Objectives*: Dry needling (DN) is a technique that involves inserting a thin filament needle through the skin to target myofascial trigger points for the treatment of musculoskeletal pain and dysfunction. Despite its efficacy in a broad plethora of musculoskeletal pain conditions, its safety remains a topic of debate among clinicians and researchers. The aim of this study was to provide an overview of the current practice of DN through a national survey, focusing on the frequency of its use and the incidence of adverse events (AEs), considering factors including physiotherapist experience, clinical workload, the extent of training received by practitioners, and the use of ultrasound guidance. *Materials and Methods*: An online cross-sectional survey was conducted. Respondents were licensed physical therapists (PTs) working in Spain. The survey covered demographics, professional data, frequency of adverse effects, and if they use ultrasound routinary for guiding interventions. *Results*: A total of 422 PTs participated in the study, mostly having 21–60 h of DN training (38.6%), less than 2 years of experience (36%), and not using ultrasound during the interventions (85.5%). Post-needling soreness and bent needles were the most common AEs, with most severe events rarely reported. Adverse event frequencies varied significantly based on training hours, experience, patient percentage treated with DN, and weekly clinical dedication. Clinicians with more hours of DN training or fewer years of experience reported higher incidences of certain complications. *Conclusions*: DN is a common intervention among PTs, with minor AEs frequently occurring and major AEs being less common but still significant. The accidental puncture of non-desired structures highlights the necessity for improve training on anatomical landmarks, needle insertion depth, cross-sectional anatomy education, and patient monitoring. To ensure safe practice, emphasize comprehensive training, adhere to safety protocols, exercise caution, and prioritize the use of ultrasound-guide is encouraged.

## 1. Introduction

Dry needling (DN) is a technique that involves inserting a thin filament needle through the skin to target myofascial trigger points (TrPs) for the treatment of musculoskeletal pain and dysfunction [1]. It employs fine, solid filiform needles to penetrate myofascial TrPs, muscles, and connective tissues without administering any substances. DN is primarily used to alleviate muscle pain, reduce disability, and restore range of motion by addressing TrPs [2]. DN is applied for treating various musculoskeletal conditions, including chronic neck pain, low back pain, shoulder pain, and tension-type headaches as research indicates that DN may lead to significant reductions in pain and disability [3,4].

Despite these benefits, DN’s safety remains a topic of debate among clinicians and researchers [5]. Side effects are relatively common, ranging from mild to severe [6]. While severe adverse events (AEs) are uncommon, mild and temporary side effects such as post-needling soreness and hematoma are reported in 19–36% of patients [7,8]. Severe AEs are more likely when physical therapists (PTs) lack sufficient training in invasive techniques [6]. Serious complications, such as pneumothorax, spinal cord injury, and infections, are rare, occurring in fewer than 0.1% of cases [7,9,10]. Pneumothorax is the most frequently reported severe AE, particularly when needles are inserted deeply into paraspinal muscles like the iliocostalis, upper trapezius, and levator scapulae [11].

Furthermore, there is inconsistency in reporting DN-related AEs in randomized clinical trials [12]. This inconsistency has led organizations such as the UK’s Chartered Society of Physiotherapy (CSP) to exclude coverage for needling treatments in the thoracic region due to associated risks. Starting on 1 July 2024, the CSP will no longer cover claims for thoracic needling, though practitioners can still perform these procedures if they have proper training and an alternative insurance [13].

Despite ongoing clinical debate over DN safety, there is a lack of comprehensive research on the subject. Therefore, this study aimed to provide an overview of the current practice of DN in Spain through a national survey, focusing on the frequency of its use and the incidence of AEs considering factors including practitioner experience, clinical workload, the extent of training received by practitioners, and the use of ultrasound guidance.

## 2. Materials and Methods

### 2.1. Study Design

This study utilized an online, cross-sectional descriptive survey, adhering to the CHERRIES checklist for reporting Internet E-Surveys. The protocol received approval from a Local Ethics Committee. An informed consent document was provided, detailing the survey duration (approximately 15 min), data storage methods, researcher identities, and the study’s purpose. Participants were informed that their collegiate number from the autonomous Spanish Physiotherapy School was required and collected as personal information to prevent duplicate responses; only the principal researcher had the password to access the survey data for security purposes. By submitting their surveys after reviewing the informed consent in the first step, participants consented to the use of their data.

### 2.2. Study Population and Sample Size Estimation

The target population consisted of licensed PTS members from one of the autonomous Spanish Physiotherapy Schools. Sample size calculations were conducted using the formula $n=\frac{t^{2}\times p\times q}{d^{2}}$, with t representing the value for the selected alpha level of 0.025 in each tale ($t^{2}$ = 1.96) and p × q as the estimated variance (p×q = 0.25). For the physiotherapy population in Spain in 2023 (N = 68.838 [14]), a minimum of 382 responses was required. Participants were contacted via email and provided with a list of 84 private physiotherapy centers, 13 public and private hospitals, and 8 public primary health care centers in Spain. The survey link was distributed from January 2019 to April 2024, participation was voluntary, and no incentives were offered for participation.

### 2.3. Survey Development

The survey instrument was developed in two stages. Initially, the principal investigator created a Spanish version during previous research [14,15], which was then refined to address issues with information integration and comprehension. A final open survey version was created using Google Forms, incorporating feedback from the earlier draft. A trial with 15 participants was conducted to evaluate its usability and technical functionality before the main study began.

The survey consisted of five sections, each presented on a separate page. The first section introduced the survey, outlining its purpose and including a consent statement, indicating that participants agreed to take part in the study by completing the survey. The second section gathered demographic (age and gender) and professional information (hours of training in DN, years of experience using DN, working hours per week and the percentage of patients to whom DN is applied). The third section focused on how frequent the surveyed participants produce pain-related AEs (how often they produce post-needling soreness, symptoms worsening and DN interruption due to pain intolerance), complications related to needle defects (how often they bend, broke and loose needles and how frequent they suffer accidental self-needling), side effects of DN (how frequent they produce pneumothorax, bleeding, hematoma, accidental nerve or visceral puncture, myoedema, vegetative reactions, allergic reactions and infections). The third section assessed the frequency of pain-related AEs using a 5-point Likert scale. The scale responses ranged from Never (0%), Rarely (less than 10%), Sometimes (10% to 30%), Often (30% to 60%), to Frequently (greater than 60%). Participants reported how frequent they produced pain-related AEs (frequency of post-needling soreness, symptom worsening, or the need to interrupt DN due to pain intolerance), complications related to needle defects (the frequency of bent, stuck, broken, or lost needles, as well as incidents of accidental self-needling), the occurrence of DN side effects (pneumothorax, bleeding, hematoma, accidental nerve or visceral puncture, myoedema, fainting, allergic reactions, and infections). Finally, the last section asked the participants to indicate if they normally use ultrasound imaging during DN procedures and a blank space to clarify any response if needed or provide additional details. All questions were mandatory, and a “Back” button was available to allow participants to revise their answers if needed.

### 2.4. Statistical Analysis

Data collected from Google Forms were imported into SPSS software (Version 21 for Mac OS) for analysis. Descriptive statistics were used to summarize the data, presenting counts and percentages for categorical variables and means and standard deviations for continuous variables, along with a 95% confidence interval. Descriptive analyses were performed for each survey question, presenting counts and percentages for categorical variables, means with standard deviations for continuous variables, and qualitative assessments for the free-text responses. These analyses were conducted for the total sample as well as by categories (hours of training, years of clinical experience, percentage of patients treated with DN, and weekly dedication). To assess differences in response distributions between categories within each classification, Chi-squared tests were employed. The significance level was set at *p* < 0.05 for all statistical tests.

## 3. Results

A total of 422 physical therapists (65.9% females) participated in the study. The total number of PTs who viewed the invitation remains unknown due to the dissemination method employed (as the method used did not track or record the specific reach or engagement of the invitation). The demographic and professional data are available in Table 1. In summary, most participants reported 21–60 h of DN training (38.6%), had less than 2 years of experience (36%), used DN in 20–39% of their practice (34.6%), dedicated 31–40 h per week to clinical practice (44.5%), and did not use ultrasound to guide interventions (85.5%). On the other hand, the least represented groups included those with 61–100 h of DN training (16.1%), had over 10 years of experience (5.7%), and used DN in 80–100% of their practice (1.7%). The least common weekly dedication was under 10 h (5.9%). Only 14.5% of participants routinely used ultrasound for DN interventions.

Responses assessing painful AEs are summarized in Table 2. Post-needling soreness was the most common adverse event. Most of the respondents declared that treatment interruption due to pain intolerance, and worsening baseline pain symptoms occurred rarely.

Response distribution varied significantly based on hours of training, years of clinical experience, the percentage of patients treated with DN, and weekly dedication (*p* < 0.05), except years of clinical experience for treatment interruption and worsening of baseline pain symptoms.

Responses assessing needle-related AEs are summarized in Table 3. Bent needles were the most reported AEs, with most participants reporting that stuck, broken, and lost needles occurred rarely or never. Similarly, accidental self-needling was mostly reported as a rare event. The distribution of responses varied significantly based on hours of training, years of clinical experience, the percentage of patients treated with DN, and weekly dedication (*p* < 0.05). However, weekly dedication to clinical practice did not significantly influence the occurrence of bent needles (*p* > 0.05), while years of experience had no significant impact on lost needles (*p* > 0.05).

Responses assessing various AEs related to DN are summarized in Table 4. Pneumothorax was the least reported event, with most participants indicating it never occurred (91.8%), while hematomas and excessive bleeding were more commonly reported, with 44.8% and 40.3% of respondents indicating these events occurred rarely. Most respondents also reported that accidental nerve punctures and infections were infrequent, with 47% and 92.1% stating they never happened, respectively. The distribution of responses varied significantly based on hours of training, years of clinical experience, the percentage of patients treated with DN, and weekly dedication (*p* < 0.05), particularly for hematomas, excessive bleeding, and accidental nerve punctures. However, no significant differences were found among years of clinical experience classifications and pneumothorax or hematoma incidents.

Although specific responses for each question and group category are available in the Supplementary Materials File S1, in general, practitioners with 20–60 h of training reported more frequent bent needles, while those with over 100 h experienced excessive bleeding and worsening symptoms. Clinicians with less than 2 years of experience had more cases of hematoma and treatment interruptions, while those with 6–10 years reported higher rates of self-needling. Additionally, treating 0–19% of patients with DN was associated with more lost needles and pneumothorax, while those treating 20–39% had higher incidences of self-needling and infections. Weekly dedication of over 40 h was linked to more stuck needles, while the 31–40 h group saw increased rates of hematoma, excessive bleeding, and treatment interruptions. Symptoms worsening was most reported by those with over 100 h of training.

## 4. Discussion

This survey provides a comprehensive overview of the current state of DN in Spain. Firstly, this report analyzes the frequency of DN application among practitioners, the physical therapists level of training, and their overall experience in the field (essential for assessing the competence and readiness of practitioners to perform DN effectively) and secondly, the survey examines the incidence of pain-related AEs, complications related to needle defects, and potential side effects associated with DN (this analysis highlights areas that may require further attention or improvement for the routinary clinical practice and future trainings). By identifying how often practitioners encounter these issues, the study highlights areas that may require further attention or improvement in practice. The results indicate that DN is a widely utilized intervention among physical therapists, with both minor (52–95%) and major AEs (4.8–62%) being reported frequently.

### 4.1. Minor Adverse Events

Previous findings indicate that mild or minor AEs occur in approximately 19% to 36% of cases, making them relatively common [8]. Minor AEs were experienced by between 52 and 95% throughout a physiotherapist’s career. Our results reported a higher rate in comparison with previous studies that reported that the prevalence of minor AEs varies from 0.32% to 39.6%, depending on the specific type of event, with pain during treatment being the most frequently reported [8].

Pain after treatment was reported by 95.5% of respondents, and bruising was reported by 85.3%. These findings are in line with previous studies, such as Brady et al. [7] and Gattie et al. [8] who reported the highest rate in these minor AEs; however, our results are significantly greater. While these minor AEs are generally transient and self-limiting, they can impact patient comfort and satisfaction. Clinicians should inform patients about the possibility of these side effects to manage expectations and enhance the therapeutic relationship, especially pain after treatment and adverse events that occurred often (47.9%) or sometimes (31.3%).

### 4.2. Major Adverse Events

The occurrence of major AEs is of significant concern due to their potential impact on patient health and safety. In contrast, severe or major AEs have been documented at rates ranging from 0.01% to 0.87% per treatment [7,15], accounting for about 13% of total AEs [16]. Major AEs can occur in 0–15% of cases during a physiotherapist’s career, with syncope or symptom exacerbation (15%) being the most common, typically resulting in transient reactions that do not require medical intervention. However, there are other significant health risks, such as subdural hematoma (1.9%), pneumothorax (1.2%), nerve injuries (0.7%), and infections (0.7%) [8], which may require further treatment [8]. Our results about major AEs, including prolonged aggravation of symptoms, accidental nerve puncture, and fainting, were also reported at notable rates.

Previous surveys [7,8,15,16] reported mild AEs, including bleeding, bruising, and pain during or after DN procedures. Major AEs encompassed cases of subdural hematoma, pneumothorax, nerve injuries, syncope, and forgotten needles, though these occurrences were less frequent. Notably, Gattie et al. [8] reported a relatively high prevalence of 0.7–1.9% for subdural hematoma, pneumothorax, infections, and nerve injuries. In our study, prolonged aggravation of symptoms was the most reported adverse event (62.0% of respondents), accidental nerve puncture occurred in 53.0%, and fainting was reported by 51.7%. The incidence of pneumothorax was reported by 8.2% of therapists, which is notably higher than rates documented in previous studies [7,8,15,16]. However, the data collected were obtained over the careers of physiotherapists, rather than the total number of interventions performed, as with Gattie et al. [8], suggesting that the selection of practitioners may have influenced the outcomes. Therefore, it is crucial to consider these potential risks to minimize complications during DN interventions.

On the other hand, among the reported major AEs, pneumothorax is the most frequently documented in DN case reports, with eight instances found after literature search [9,11,17,18,19,20,21]. Although hemothorax and hemopneumothorax are rare, they are serious AEs, with only two cases reported in the previous literature [22,23]. Clinicians should be aware that 64–85% of patients with pneumothorax experience acute pleuritic chest pain radiating to the arm, highlighting the importance of early recognition [24]. The most common symptoms reported in case studies included acute thoracic pain and dyspnea [9,11,17,19,20,21,22,23]. The higher prevalence of pneumothorax may be associated with needling techniques in high-risk anatomical regions, such as the thoracic area, particularly involving muscles like the rhomboid, levator scapulae [22], or iliocostalis [21], which increases the risk associated with needle placement near the pleural cavity [5]. Using shorter needles (2.5 cm) in thoracic muscles may help reduce the likelihood of pleural injury [25]. Most reported cases showed complete recovery, although some AEs required invasive procedures [17,18,20,22,23], while others resolved with conservative management, such as oxygen therapy [9,11,19,21].

Deep infections were the second most frequently reported AE after pneumothorax in case reports, with three documented instances [10,26,27]. These infections primarily occurred in the knee [10,26] and spine regions [27], particularly in patients with implants or a history of surgical interventions. Symptoms often included pain, swelling, or tenderness. Prompt diagnosis and intervention are critical, as these infections can lead to significant morbidity [28]. Implementing infection prevention strategies—such as maintaining proper hand and environmental hygiene, assessing clinical history for infection risks (e.g., prior surgeries), using personal protective equipment (e.g., gloves), and applying skin antiseptics (e.g., alcohol and chlorhexidine sprays)—can significantly reduce the risk of deep infections during DN [29]. These strategies are essential for minimizing pathogen transmission and ensuring patient safety.

The literature reflects that nerve injury following DN is exceptionally rare, with only one case reported for the radial nerve [30]. Our study did not register nerve injury cases, but a great proportion of respondents documented accidental puncture of nerves (53%). Gattie et al. [8] reported 1.2% of cases of nerve injury among PTs. This could be in relation with accidental puncture, which did not imply a nerve injury, and most of the cases of interruption of the technique or changes regarding the region of DN is sufficient to avoid a worse event. However, injury to nerves caused by DN can be severe, for example, in one reported case, the patient did not regain function or recover from disability after one year of follow-up [30]. Clinicians should familiarize themselves with regional neuroanatomy and consider using shorter needles to minimize the risk of nerve injury.

### 4.3. Clinical Implications

DN appears to be an integral component of physical therapy practice for many clinicians. A significant proportion of therapists applied DN to a substantial percentage of their patients, with 34.6% treating 20–39% of their patient population. This widespread use aligns with the growing acceptance and integration of DN into musculoskeletal pain management [8]. The diversity in experience and training among respondents suggests that DN is practiced across a broad spectrum of clinicians, from recent graduates to seasoned practitioners.

The survey encompassed 422 physical therapists with varying levels of experience and training in DN. Despite a considerable amount of training, 79.6% had more than 20 h of instruction, and both minor and major AEs were commonly reported. Despite a high percentage of respondents having substantial training in DN, the occurrence of AEs suggests that the amount of training may not be sufficient to ensure safety. According with our results, clinicians with higher hours of DN training did not consistently report fewer adverse effects; in some cases, they actually reported more. The data show that clinicians with more training hours often encountered higher rates of certain AEs. In fact, therapists with more training and experience reported higher occurrences of major AEs. This paradox may be attributed to increased exposure over time; more experienced therapists likely perform DN more frequently, thereby increasing the likelihood of encountering AEs [8]. Specifically, clinicians with extensive DN training (over 100 h) reported increased occurrences of complications such as excessive bleeding and symptom aggravation. Therefore, while more training might contribute to technical skill, it does not necessarily correlate with fewer AEs, likely because more trained clinicians tend to apply DN more frequently and with greater depth or in complex cases, which can elevate AE risks. This underscores the importance of not only training volume but also training content focused on safety protocols and risk management. These findings underscore the necessity for continuous professional development, focusing not just on technical skills but also on risk management and patient safety.

Ultrasound-guided interventions were demonstrated to be the safest option since it improves significantly the accuracy of needle placement, especially within the high-risk group of muscles [31]. The low use of ultrasound guidance among respondents is notable, especially considering recommendations that ultrasound can enhance accuracy and reduce risks associated with DN [5,32]. Ultrasound imaging allows clinicians to visualize underlying structures, aiding in the avoidance of critical anatomical features such as nerves and organs. Increased adoption of ultrasound guidance could potentially decrease the incidence of both minor and major AEs. Barriers to utilization, such as cost, lack of training, or limited access to equipment, should be addressed through institutional support and professional education initiatives.

Alternatively, there are studies assessing how landmarks and anthropometric references can be introduced in regression models to calculate the most appropriate needle length and assist clinicians in avoiding AE associated with specific anatomical structures [33,34,35,36]. However, it should be noted that these statistical models are limited to few structures and there is a high variability in the explained variance depending on the structure. For these reasons, developing regression models for different high-risk structures is encouraged for future studies to elucidate whether blinded interventions are safe [37,38,39] or should be avoided [40,41].

When compared to other interventions for musculoskeletal pain, DN appears to have a similar or lower risk profile. Spinal manipulation, for example, has been associated with minor AEs in up to 60.9% of patients and carries risks of serious complications such as vertebral artery dissection [42,43]. Pharmacological treatments like opioids and non-steroidal anti-inflammatory drugs are associated with higher rates of AEs, including dependency, gastrointestinal bleeding, and cardiovascular events [44,45]. These comparisons suggest that while DN is not risks free, it may be a comparatively safer alternative to some other common treatments.

### 4.4. Limitations

The reliance on self-reported data may introduce recall bias, as therapists who have experienced AEs might be more inclined to participate, potentially leading to overreporting. The cross-sectional design captures a snapshot in time and does not allow for assessment of causality or changes over time. Additionally, the sample may not represent all physical therapists, limiting the generalizability of the findings. The lack of detailed context surrounding AEs, such as specific techniques used or patient characteristics, limits the ability to identify specific risk factors.

### 4.5. Future Research

Further research is needed to explore the factors contributing to AEs in DN more comprehensively. Longitudinal studies could assess the impact of training interventions, practice changes, and the adoption of ultrasound guidance over time. Additionally, qualitative research exploring therapists’ experiences and perceptions could provide deeper insights into barriers and facilitators to safe practice. Investigating patient outcomes and satisfaction in relation to AEs would also be valuable for informing clinical practice and enhancing patient-centered care.

Future research should prioritize the establishment of a national, centralized database for reporting AEs related to intervention, capturing detailed information on side effects and contributing preconditions. This database could facilitate the collection of hard data, thereby strengthening the reliability of findings over participant recollection alone. Such a system, modeled after existing frameworks in other countries, would support a more robust analysis of AEs and contribute valuable insights into preventive measures. Additionally, incorporating findings from this database into training curricula could enhance clinical practices, helping practitioners recognize and address potential risk factors more effectively.

## 5. Conclusions

DN is a prevalent intervention among PTs, with minor AEs being frequent and major AEs less common but significant. Accidental nerve puncture and its associated complications highlight the need for enhanced training focused on anatomical landmarks, needle insertion depth, and patient monitoring. The findings underscore the necessity for comprehensive training, adherence to safety protocols, and cautious practice, especially in high-risk areas. Incorporating ultrasound guidance and developing standardized guidelines may enhance safety and reduce the incidence of AEs. Ongoing education, patient communication, and systematic monitoring are essential components in advancing the safe practice of DN.

## Figures and Tables

**Table 1:** Demographic and professional data of respondents.

Surveyed Physiotherapists (*n* = 422)	
Demographic information	
Age (years), mean ± SD	31.26 ± 6.31
Males, *n* (%)	144 (34.1%)
Hours of Training	
0–20 h, *n* (%)	86 (20.4%)
21–60 h, *n* (%)	163 (38.6%)
61–100 h, *n* (%)	68 (16.1%)
+100 h, *n* (%)	105 (24.9%)
Professional Experience	
<2 years, *n* (%)	152 (36%)
3–5 years, *n* (%)	140 (33.2%)
5–10 years, *n* (%)	106 (25.1%)
+10 years, *n* (%)	24 (5.7%)
Percentage of use	
0–19%, *n* (%)	132 (31.5%)
20–39%, *n* (%)	145 (34.6)
40–59%, *n* (%)	81 (19.3%)
60–79%, *n* (%)	54 (12.9%)
80–100%, *n* (%)	7 (1.7%)
Weekly dedication	
<10 h, *n* (%)	25 (85.9%)
11–20 h, *n* (%)	40 (9.5%)
21–30 h, *n* (%)	89 (21.1%)
31 + 40 h, *n* (%)	188 (44.5%)
>40 h, *n* (%)	80 (19%)
Routinary use of ultrasound for guiding interventions	
Yes, *n* (%)	61 (14.5%)
No, *n* (%)	361 (85.5%)

**Table 2:** How often do you produce the following pain-related adverse events?

	Surveyed Physiotherapists (*n* = 422)	Group Distribution Differences			
		Hours of Training	Years of Clinical Experience	Percentage of Patients Treated Using DN	Weekly Dedication
Post-needling soreness					
Never	12 (2.8%)	*p* = 0.014	*p* = 0.008	*p* = 0.037	*p* < 0.001
Rarely	33 (7.8%)				
Sometimes	132 (31.3%)				
Often	202 (47.9%)				
Frequently	43 (10.2%)				
Treatment interruption due to pain intolerance					
Never	86 (20.4%)	*p* = 0.002	0.173	*p* < 0.001	*p* < 0.001
Rarely	243 (57.6%)				
Sometimes	84 (19.9%)				
Often	5 (1.2%)				
Frequently	2 (0.5%)				
Worsening the baseline pain symptoms					
Never	160 (37.9%)	*p* = 0.013	0.100	*p* < 0.001	*p* = 0.005
Rarely	232 (55%)				
Sometimes	23 (5.5%)				
Often	7 (1.7%)				
Frequently	0 (0%)				

**Table 3:** How often the following needle defects complications happened to you?

	Surveyed Physiotherapists (*n* = 422)	Group Distribution Differences			
		Hours of Training	Years of Clinical Experience	Percentage of Patients Treated Using DN	Weekly Dedication
Bent needles					
Never	130 (31%)	*p* < 0.001	*p* < 0.001	*p* < 0.001	*p* = 0.296
Rarely	161 (38.3%)				
Sometimes	113 (26.9%)				
Often	16 (3.8%)				
Frequently	0 (0%)				
Stuck needles					
Never	198 (47.1%)	*p* = 0.003	*p* < 0.001	*p* = 0.002	*p* = 0.002
Rarely	155 (36.9%)				
Sometimes	67 (16%)				
Often	0 (0%)				
Frequently	0 (0%)				
Broken needles					
Never	400 (95.2%)	*p* = 0.030	*p* < 0.001	*p* = 0.014	*p* < 0.001
Rarely	20 (4.8%)				
Sometimes	0 (0%)				
Often	0 (0%)				
Frequently	0 (0%)				
Lost needles					
Never	198 (47.1)	*p* = 0.014	*p* = 0.256	*p* < 0.001	*p* = 0.009
Rarely	148 (35.2)				
Sometimes	67 (16.0)				
Often	7 (1.7)				
Frequently	0 (0.0)				
Accidental self-needling					
Never	222 (52.6)	*p* < 0.001	*p* = 0.010	*p* < 0.001	*p* = 0.029
Rarely	182 (43.1)				
Sometimes	16 (3.8)				
Often	0 (0.0)				
Frequently	0 (0.0)				

**Table 4:** How often do you provoke the following adverse events during or after dry needling interventions?

	Pneumothorax	Hematoma	Excessive Bleeding	Accidental Nerve Puncture	Accidental Visceral Puncture	Infection	Fainting	Allergy	Myoedema
Scores									
Never, *n* (%)	380 (91.8%)	62 (14.7%)	241 (57.1%)	196 (47%)	387 (92.1%)	387 (92.1%)	203 (48.3%)	357 (86.2%)	90 (21.5%)
Rarely, *n* (%)	32 (7.7%)	189 (44.8%)	170 (40.3%)	161 (38.6%)	33 (7.9%)	33 (7.9%)	189 (44.8%)	57 (13.8%)	188 (45%)
Sometimes, *n* (%)	2 (0.5%)	158 (37.4%)	7 (1.7%)	60 (14.4%)	0 (0%)	0 (0%)	28 (6.6%)	2 (0.5%)	123 (29.4%)
Often, *n* (%)	0 (0%)	13 (3.1%)	0 (0%)	0 (0%)	0 (0%)	0 (0%)	0 (0%)	0 (0%)	17 (4.1%)
Frequently, *n* (%)	0 (0%)	0 (0%)	0 (0%)	0 (0%)	0 (0%)	0 (0%)	0 (0%)	0 (0%)	0 (0%)
Hours of Training									
	*p* < 0.001	*p* < 0.001	*p* < 0.001	*p* = 0.002	*p* < 0.001	*p* = 0.231	0.591	*p* = 0.012	*p* = 0.096
Years of Clinical Experience									
	*p* = 0.431	*p* = 0.286	*p* = 0.015	*p* = 0.015	*p* = 0.025	*p* = 0.002	*p* < 0.001	*p* = 0.004	*p* < 0.001
Percentage of patients treated using DN									
	*p* = 0.066	*p* = 0.002	*p* = 0.012	*p* = 0.002	*p* = 0.024	*p* < 0.001	*p* < 0.001	*p* = 0.181	*p* < 0.001
Weekly dedication									
	*p* = 0.024	*p* < 0.001	*p* = 0.010	*p* = 0.036	*p* = 0.001	*p* < 0.001	*p* = 0.001	*p* < 0.001	*p* < 0.001

## Data Availability

Data are contained within the article or Supplementary Materials.

## Supplementary Materials

The following supporting information can be downloaded at: https://www.mdpi.com/article/10.3390/medicina60111869/s1, File S1: Responses for each question by group category.
