# Supplementary material for: Current State of Dry Needling Practices: A Comprehensive Analysis on Use, Training, and Safety
Source: Medicina (Kaunas). 2024 Nov 14;60(11):1869. doi: 10.3390/medicina60111869 (PMC11596814; doi:10.3390/medicina60111869)
Supplement: Supplementary file 1 [file medicina-60-01869-s001.zip › medicina-3302665-supplementary.pdf]

Crosstabs

Notes

Output Created		14-OCT-2024 19:53:07
Comments		
Input	Data	/Users/juanantoniovaleracalero/Library/CloudStorage/OneDrive-UniversidadComplutensedeMadrid(UCM)/Investigación/Encuestapunción/SPSS Encues EA.sav
	Active Dataset	ConjuntoDatos1
	Filter	<none>
	Weight	<none>
	Split File	<none>
	N of Rows in Working Data File	422
Missing Value Handling	Definition of Missing	User-defined missing values are treated as missing.
	Cases Used	Statistics for each table are based on all the cases with valid data in the specified range(s) for all variables in each table.
Syntax		CROSSTABS<br>/TABLES=Postneedling_Soreness DN_Interruption Symptoms_Worsening Bent_Needle Stuck_Needle<br>Broken_Needle Lost_Needle Self_Needling Pneumothorax Hematoma Excessive_Bleeding<br>Accidental_Nerve_Puncture Accidental_Visceral_Puncture Infection Fainting Allergy Myoedema BY<br>Hours_of_Training Professional_Experience Percentage_of_DN_use Weekly_Dedication<br>/FORMAT=AVALUE TABLES<br>/STATISTICS=CHISQ<br>/CELLS=ROW<br>/COUNT ROUND CELL.
Resources	Processor Time	00:00:00,28
	Elapsed Time	00:00:00,00

Notes

	Dimensions Requested	2
	Cells Available	524245

Case Processing Summary

	Cases					
	Valid		Missing		Total	
	N	Percent	N	Percent	N	Percent
Postneedling_Soreness * Hours_of_Training	422	100.0%	0	0.0%	422	100.0%
Postneedling_Soreness * Professional_Experience	422	100.0%	0	0.0%	422	100.0%
Postneedling_Soreness * Percentage_of_DN_use	419	99.3%	3	0.7%	422	100.0%
Postneedling_Soreness * Weekly_Dedication	422	100.0%	0	0.0%	422	100.0%
DN_Interruption * Hours_of_Training	420	99.5%	2	0.5%	422	100.0%
DN_Interruption * Professional_Experience	420	99.5%	2	0.5%	422	100.0%
DN_Interruption * Percentage_of_DN_use	417	98.8%	5	1.2%	422	100.0%
DN_Interruption * Weekly_Dedication	420	99.5%	2	0.5%	422	100.0%
Symptoms_Worsening * Hours_of_Training	422	100.0%	0	0.0%	422	100.0%
Symptoms_Worsening * Professional_Experience	422	100.0%	0	0.0%	422	100.0%
Symptoms_Worsening * Percentage_of_DN_use	419	99.3%	3	0.7%	422	100.0%
Symptoms_Worsening * Weekly_Dedication	422	100.0%	0	0.0%	422	100.0%
Bent_Needle * Hours_of_Training	420	99.5%	2	0.5%	422	100.0%
Bent_Needle * Professional_Experience	420	99.5%	2	0.5%	422	100.0%
Bent_Needle * Percentage_of_DN_use	417	98.8%	5	1.2%	422	100.0%
Bent_Needle * Weekly_Dedication	420	99.5%	2	0.5%	422	100.0%
Stuck_Needle * Hours_of_Training	420	99.5%	2	0.5%	422	100.0%
Stuck_Needle * Professional_Experience	420	99.5%	2	0.5%	422	100.0%
Stuck_Needle * Percentage_of_DN_use	417	98.8%	5	1.2%	422	100.0%
Stuck_Needle * Weekly_Dedication	420	99.5%	2	0.5%	422	100.0%
Broken_Needle * Hours_of_Training	420	99.5%	2	0.5%	422	100.0%

Case Processing Summary

	Cases					
	Valid		Missing		Total	
	N	Percent	N	Percent	N	Percent
Broken_Needle * Professional_Experience	420	99.5%	2	0.5%	422	100.0%
Broken_Needle * Percentage_of_DN_use	417	98.8%	5	1.2%	422	100.0%
Broken_Needle * Weekly_Dedication	420	99.5%	2	0.5%	422	100.0%
Lost_Needle * Hours_of_Training	420	99.5%	2	0.5%	422	100.0%
Lost_Needle * Professional_Experience	420	99.5%	2	0.5%	422	100.0%
Lost_Needle * Percentage_of_DN_use	417	98.8%	5	1.2%	422	100.0%
Lost_Needle * Weekly_Dedication	420	99.5%	2	0.5%	422	100.0%
Self_Needling * Hours_of_Training	420	99.5%	2	0.5%	422	100.0%
Self_Needling * Professional_Experience	420	99.5%	2	0.5%	422	100.0%
Self_Needling * Percentage_of_DN_use	417	98.8%	5	1.2%	422	100.0%
Self_Needling * Weekly_Dedication	420	99.5%	2	0.5%	422	100.0%
Pneumothorax * Hours_of_Training	414	98.1%	8	1.9%	422	100.0%
Pneumothorax * Professional_Experience	414	98.1%	8	1.9%	422	100.0%
Pneumothorax * Percentage_of_DN_use	411	97.4%	11	2.6%	422	100.0%
Pneumothorax * Weekly_Dedication	414	98.1%	8	1.9%	422	100.0%
Hematoma * Hours_of_Training	422	100.0%	0	0.0%	422	100.0%
Hematoma * Professional_Experience	422	100.0%	0	0.0%	422	100.0%
Hematoma * Percentage_of_DN_use	419	99.3%	3	0.7%	422	100.0%
Hematoma * Weekly_Dedication	422	100.0%	0	0.0%	422	100.0%
Excessive_Bleeding * Hours_of_Training	418	99.1%	4	0.9%	422	100.0%
Excessive_Bleeding * Professional_Experience	418	99.1%	4	0.9%	422	100.0%
Excessive_Bleeding * Percentage_of_DN_use	415	98.3%	7	1.7%	422	100.0%
Excessive_Bleeding * Weekly_Dedication	418	99.1%	4	0.9%	422	100.0%

Case Processing Summary

	Cases					
	Valid		Missing		Total	
	N	Percent	N	Percent	N	Percent
Accidental_Nerve_Puncture * Hours_of_Training	417	98.8%	5	1.2%	422	100.0%
Accidental_Nerve_Puncture * Professional_Experience	417	98.8%	5	1.2%	422	100.0%
Accidental_Nerve_Puncture * Percentage_of_DN_use	414	98.1%	8	1.9%	422	100.0%
Accidental_Nerve_Puncture * Weekly_Dedication	417	98.8%	5	1.2%	422	100.0%
Accidental_Visceral_Puncture * Hours_of_Training	420	99.5%	2	0.5%	422	100.0%
Accidental_Visceral_Puncture * Professional_Experience	420	99.5%	2	0.5%	422	100.0%
Accidental_Visceral_Puncture * Percentage_of_DN_use	417	98.8%	5	1.2%	422	100.0%
Accidental_Visceral_Puncture * Weekly_Dedication	420	99.5%	2	0.5%	422	100.0%
Infection * Hours_of_Training	416	98.6%	6	1.4%	422	100.0%
Infection * Professional_Experience	416	98.6%	6	1.4%	422	100.0%
Infection * Percentage_of_DN_use	413	97.9%	9	2.1%	422	100.0%
Infection * Weekly_Dedication	416	98.6%	6	1.4%	422	100.0%
Fainting * Hours_of_Training	420	99.5%	2	0.5%	422	100.0%
Fainting * Professional_Experience	420	99.5%	2	0.5%	422	100.0%
Fainting * Percentage_of_DN_use	417	98.8%	5	1.2%	422	100.0%
Fainting * Weekly_Dedication	420	99.5%	2	0.5%	422	100.0%
Allergy * Hours_of_Training	414	98.1%	8	1.9%	422	100.0%
Allergy * Professional_Experience	414	98.1%	8	1.9%	422	100.0%
Allergy * Percentage_of_DN_use	411	97.4%	11	2.6%	422	100.0%
Allergy * Weekly_Dedication	414	98.1%	8	1.9%	422	100.0%
Myoedema * Hours_of_Training	418	99.1%	4	0.9%	422	100.0%
Myoedema * Professional_Experience	418	99.1%	4	0.9%	422	100.0%

Case Processing Summary

	Cases					
	Valid		Missing		Total	
	N	Percent	N	Percent	N	Percent
Myoedema * Percentage_of_DN_use	415	98.3%	7	1.7%	422	100.0%
Myoedema * Weekly_Dedication	418	99.1%	4	0.9%	422	100.0%

Postneedling_Soreness * Hours_of_Training

Crosstab

% within Postneedling_Soreness

		Hours_of_Training			
		0-20 hours	20-60 hours	80-100 hours	>100 hours
Postneedling_Soreness	Never	66.7%	33.3%		
	Rarely	24.2%	33.3%	18.2%	24.2%
	Sometimes	19.7%	34.1%	20.5%	25.8%
	Often	18.8%	40.6%	13.4%	27.2%
	Always	14.0%	48.8%	18.6%	18.6%
Total		20.4%	38.6%	16.1%	24.9%

Crosstab

% within Postneedling_Soreness

		Total
Postneedling_Soreness	Never	100.0%
	Rarely	100.0%
	Sometimes	100.0%
	Often	100.0%
	Always	100.0%
Total		100.0%

Chi-Square Tests

	Value	df	Asymptotic Significance (2-sided)
Pearson Chi-Square	25.231[a]	12	.014
Likelihood Ratio	25.419	12	.013
Linear-by-Linear Association	2.680	1	.102
N of Valid Cases	422		

a. 4 cells (20.0%) have expected count less than 5. The minimum expected count is 1.93.

Postneedling_Soreness * Professional_Experience

Crosstab

% within Postneedling_Soreness

		Professional_Experience				
		<2 years	3-5 years	6-10 years	>10 years	Total
Postneedling_Soreness	Never	33.3%	16.7%	25.0%	25.0%	100.0%
	Rarely	30.3%	54.5%	6.1%	9.1%	100.0%
	Sometimes	37.9%	26.5%	30.3%	5.3%	100.0%
	Often	36.1%	32.7%	25.7%	5.4%	100.0%
	Always	34.9%	44.2%	20.9%		100.0%
Total		36.0%	33.2%	25.1%	5.7%	100.0%

Chi-Square Tests

	Value	df	Asymptotic Significance (2-sided)
Pearson Chi-Square	26.905[a]	12	.008
Likelihood Ratio	27.149	12	.007
Linear-by-Linear Association	1.462	1	.227
N of Valid Cases	422		

a. 6 cells (30.0%) have expected count less than 5. The minimum expected count is .68.

Postneedling_Soreness * Percentage_of_DN_use

Crosstab

% within Postneedling_Soreness

		Percentage_of_DN_use				
		0-19%	20-39%	40-59%	60-79%	80-100%
Postneedling_Soreness	Never	58.3%	25.0%	16.7%		
	Rarely	27.3%	57.6%	15.2%		
	Sometimes	28.7%	31.0%	18.6%	20.2%	1.6%
	Often	31.7%	35.6%	18.3%	11.9%	2.5%
	Always	34.9%	25.6%	30.2%	9.3%	
Total		31.5%	34.6%	19.3%	12.9%	1.7%

Crosstab

% within Postneedling_Soreness

		Total
Postneedling_Soreness	Never	100.0%
	Rarely	100.0%
	Sometimes	100.0%
	Often	100.0%
	Always	100.0%
Total		100.0%

Chi-Square Tests

	Value	df	Asymptotic Significance (2-sided)
Pearson Chi-Square	27.388[a]	16	.037
Likelihood Ratio	32.226	16	.009
Linear-by-Linear Association	.964	1	.326
N of Valid Cases	419		

a. 10 cells (40.0%) have expected count less than 5. The minimum expected count is .20.

Postneedling_Soreness * Weekly_Dedication

Crosstab

% within Postneedling_Soreness

		Weekly_Dedication			
		<10 hours	11-20 hours	21-30 hours	31-40 hours
Postneedling_Soreness	Never				50.0%
	Rarely		21.2%	33.3%	21.2%
	Sometimes	10.6%	12.9%	12.9%	42.4%
	Often	4.0%	6.9%	23.8%	48.5%
	Always	7.0%	4.7%	30.2%	48.8%
Total		5.9%	9.5%	21.1%	44.5%

Crosstab

% within Postneedling_Soreness

		Weekly_Dedi...	
		>40 hours	Total
Postneedling_Soreness	Never	50.0%	100.0%
	Rarely	24.2%	100.0%
	Sometimes	21.2%	100.0%
	Often	16.8%	100.0%
	Always	9.3%	100.0%
Total		19.0%	100.0%

Chi-Square Tests

	Value	df	Asymptotic Significance (2-sided)
Pearson Chi-Square	45.086[a]	16	<.001
Likelihood Ratio	49.223	16	<.001
Linear-by-Linear Association	.606	1	.436
N of Valid Cases	422		

a. 8 cells (32.0%) have expected count less than 5. The minimum expected count is .71.

DN_Interruption * Hours_of_Training

Crosstab

% within DN_Interruption

		Hours_of_Training			
		0-20 hours	20-60 hours	80-100 hours	>100 hours
DN_Interruption	Never	30.2%	46.5%	14.0%	9.3%
	Rarely	18.1%	36.2%	16.5%	29.2%
	Sometimes	19.0%	41.7%	14.3%	25.0%
	Often			40.0%	60.0%
	Always				100.0%
Total		20.5%	38.8%	15.7%	25.0%

Crosstab

% within DN_Interruption

		Total
DN_Interruption	Never	100.0%
	Rarely	100.0%
	Sometimes	100.0%
	Often	100.0%
	Always	100.0%
Total		100.0%

Chi-Square Tests

	Value	df	Asymptotic Significance (2-sided)
Pearson Chi-Square	30.523[a]	12	.002
Likelihood Ratio	33.831	12	<.001
Linear-by-Linear Association	13.813	1	<.001
N of Valid Cases	420		

a. 8 cells (40.0%) have expected count less than 5. The minimum expected count is .31.

DN_Interruption * Professional_Experience

Crosstab

% within DN_Interruption

		Professional_Experience				
		<2 years	3-5 years	6-10 years	>10 years	Total
DN_Interruption	Never	31.4%	33.7%	32.6%	2.3%	100.0%
	Rarely	37.4%	30.0%	26.3%	6.2%	100.0%
	Sometimes	34.5%	40.5%	16.7%	8.3%	100.0%
	Often	60.0%	40.0%			100.0%
	Always		100.0%			100.0%
Total		35.7%	33.3%	25.2%	5.7%	100.0%

Chi-Square Tests

	Value	df	Asymptotic Significance (2-sided)
Pearson Chi-Square	16.420[a]	12	.173
Likelihood Ratio	18.733	12	.095
Linear-by-Linear Association	.844	1	.358
N of Valid Cases	420		

a. 10 cells (50.0%) have expected count less than 5. The minimum expected count is .11.

DN_Interruption * Percentage_of_DN_use

Crosstab

% within DN_Interruption

		Percentage_of_DN_use					
		0-19%	20-39%	40-59%	60-79%	80-100%	Total
DN_Interruption	Never	44.6%	38.6%	8.4%	8.4%		100.0%
	Rarely	27.6%	32.1%	24.3%	14.4%	1.6%	100.0%
	Sometimes	33.3%	41.7%	9.5%	11.9%	3.6%	100.0%
	Often			100.0%			100.0%
	Always			100.0%			100.0%
Total		31.7%	34.8%	19.4%	12.5%	1.7%	100.0%

Chi-Square Tests

	Value	df	Asymptotic Significance (2-sided)
Pearson Chi-Square	54.131[a]	16	<.001
Likelihood Ratio	50.758	16	<.001
Linear-by-Linear Association	6.239	1	.012
N of Valid Cases	417		

a. 13 cells (52.0%) have expected count less than 5. The minimum expected count is .03.

DN_Interruption * Weekly_Dedication

Crosstab

% within DN_Interruption

		Weekly_Dedication			
		<10 hours	11-20 hours	21-30 hours	31-40 hours
DN_Interruption	Never	3.5%	7.0%	15.1%	45.3%
	Rarely	5.3%	9.9%	21.4%	43.2%
	Sometimes	6.0%	9.5%	28.6%	48.8%
	Often	40.0%			60.0%
	Always		100.0%		
Total		5.5%	9.5%	21.2%	44.8%

Crosstab

% within DN_Interruption

		Weekly_Dedi...	
		>40 hours	Total
DN_Interruption	Never	29.1%	100.0%
	Rarely	20.2%	100.0%
	Sometimes	7.1%	100.0%
	Often		100.0%
	Always		100.0%
Total		19.0%	100.0%

Chi-Square Tests

	Value	df	Asymptotic Significance (2-sided)
Pearson Chi-Square	48.961[a]	16	<.001
Likelihood Ratio	36.888	16	.002
Linear-by-Linear Association	14.614	1	<.001
N of Valid Cases	420		

a. 12 cells (48.0%) have expected count less than 5. The minimum expected count is .11.

Symptoms_Worsening * Hours_of_Training

Crosstab

% within Symptoms_Worsening

		Hours_of_Training			
		0-20 hours	20-60 hours	80-100 hours	>100 hours
Symptoms_Worsening	Never	21.3%	36.9%	21.3%	20.6%
	Rarely	17.2%	38.8%	13.8%	30.2%
	Sometimes	39.1%	43.5%	8.7%	8.7%
	Often	42.9%	57.1%		
Total		20.4%	38.6%	16.1%	24.9%

Crosstab

% within Symptoms_Worsening

		Total
Symptoms_Worsening	Never	100.0%
	Rarely	100.0%
	Sometimes	100.0%
	Often	100.0%
Total		100.0%

Chi-Square Tests

	Value	df	Asymptotic Significance (2-sided)
Pearson Chi-Square	21.021[a]	9	.013
Likelihood Ratio	23.278	9	.006
Linear-by-Linear Association	1.790	1	.181
N of Valid Cases	422		

a. 6 cells (37.5%) have expected count less than 5. The minimum expected count is 1.13.

Symptoms_Worsening * Professional_Experience

Crosstab

% within Symptoms_Worsening

		Professional_Experience				
		<2 years	3-5 years	6-10 years	>10 years	Total
Symptoms_Worsening	Never	33.1%	30.6%	31.3%	5.0%	100.0%
	Rarely	39.7%	33.6%	21.1%	5.6%	100.0%
	Sometimes	21.7%	34.8%	30.4%	13.0%	100.0%
	Often	28.6%	71.4%			100.0%
Total		36.0%	33.2%	25.1%	5.7%	100.0%

Chi-Square Tests

	Value	df	Asymptotic Significance (2-sided)
Pearson Chi-Square	14.677[a]	9	.100
Likelihood Ratio	15.584	9	.076
Linear-by-Linear Association	.481	1	.488
N of Valid Cases	422		

a. 5 cells (31.3%) have expected count less than 5. The minimum expected count is .40.

Symptoms_Worsening * Percentage_of_DN_use

Crosstab

% within Symptoms_Worsening

		Percentage_of_DN_use				
		0-19%	20-39%	40-59%	60-79%	80-100%
Symptoms_Worsening	Never	38.9%	34.4%	20.4%	6.4%	
	Rarely	27.6%	33.2%	20.3%	17.2%	1.7%
	Sometimes	21.7%	47.8%	8.7%	8.7%	13.0%
	Often	28.6%	42.9%		28.6%	
Total		31.5%	34.6%	19.3%	12.9%	1.7%

Crosstab

% within Symptoms_Worsening

		Total
Symptoms_Worsening	Never	100.0%
	Rarely	100.0%
	Sometimes	100.0%
	Often	100.0%
Total		100.0%

Chi-Square Tests

	Value	df	Asymptotic Significance (2-sided)
Pearson Chi-Square	39.653[a]	12	<.001
Likelihood Ratio	33.762	12	<.001
Linear-by-Linear Association	10.364	1	.001
N of Valid Cases	419		

a. 10 cells (50.0%) have expected count less than 5. The minimum expected count is .12.

Symptoms_Worsening * Weekly_Dedication

Crosstab

% within Symptoms_Worsening

		Weekly_Dedication			
		<10 hours	11-20 hours	21-30 hours	31-40 hours
Symptoms_Worsening	Never	3.8%	5.6%	22.5%	43.8%
	Rarely	7.3%	11.2%	22.0%	43.1%
	Sometimes	8.7%	8.7%		69.6%
	Often		42.9%	28.6%	28.6%
Total		5.9%	9.5%	21.1%	44.5%

Crosstab

% within Symptoms_Worsening

		Weekly_Dedi... >40 hours	Total
Symptoms_Worsening	Never	24.4%	100.0%
	Rarely	16.4%	100.0%
	Sometimes	13.0%	100.0%
	Often		100.0%
Total		19.0%	100.0%

Chi-Square Tests

	Value	df	Asymptotic Significance (2-sided)
Pearson Chi-Square	28.322[a]	12	.005
Likelihood Ratio	31.005	12	.002
Linear-by-Linear Association	7.006	1	.008
N of Valid Cases	422		

a. 9 cells (45.0%) have expected count less than 5. The minimum expected count is .41.

Bent_Needle * Hours_of_Training

Crosstab

% within Bent_Needle

		Hours_of_Training				Total
		0-20 hours	20-60 hours	80-100 hours	>100 hours	
Bent_Needle	Never	24.6%	47.7%	10.8%	16.9%	100.0%
	Rarely	19.9%	26.7%	23.6%	29.8%	100.0%
	Sometimes	16.8%	42.5%	9.7%	31.0%	100.0%
	Often	18.8%	62.5%	18.8%		100.0%
Total		20.5%	38.8%	15.7%	25.0%	100.0%

Chi-Square Tests

	Value	df	Asymptotic Significance (2-sided)
Pearson Chi-Square	34.864[a]	9	<.001
Likelihood Ratio	39.074	9	<.001
Linear-by-Linear Association	2.401	1	.121
N of Valid Cases	420		

a. 3 cells (18.8%) have expected count less than 5. The minimum expected count is 2.51.

Bent_Needle * Professional_Experience

Crosstab

% within Bent_Needle

		Professional_Experience				Total
		<2 years	3-5 years	6-10 years	>10 years	
Bent_Needle	Never	47.7%	31.5%	20.8%		100.0%
	Rarely	35.4%	35.4%	21.1%	8.1%	100.0%
	Sometimes	24.8%	31.9%	33.6%	9.7%	100.0%
	Often	18.8%	37.5%	43.8%		100.0%
Total		35.7%	33.3%	25.2%	5.7%	100.0%

Chi-Square Tests

	Value	df	Asymptotic Significance (2-sided)
Pearson Chi-Square	31.334[a]	9	<.001
Likelihood Ratio	38.596	9	<.001
Linear-by-Linear Association	21.767	1	<.001
N of Valid Cases	420		

a. 2 cells (12.5%) have expected count less than 5. The minimum expected count is .91.

Bent_Needle * Percentage_of_DN_use

Crosstab

% within Bent_Needle

		Percentage_of_DN_use					Total
		0-19%	20-39%	40-59%	60-79%	80-100%	
Bent_Needle	Never	39.2%	46.9%	10.0%	3.8%		100.0%
	Rarely	31.6%	31.0%	19.6%	15.8%	1.9%	100.0%
	Sometimes	22.1%	26.5%	30.1%	17.7%	3.5%	100.0%
	Often	37.5%	31.3%	18.8%	12.5%		100.0%
Total		31.7%	34.8%	19.4%	12.5%	1.7%	100.0%

Chi-Square Tests

	Value	df	Asymptotic Significance (2-sided)
Pearson Chi-Square	43.240[a]	12	<.001
Likelihood Ratio	47.844	12	<.001
Linear-by-Linear Association	22.497	1	<.001
N of Valid Cases	417		

a. 6 cells (30.0%) have expected count less than 5. The minimum expected count is .27.

Bent_Needle * Weekly_Dedication

Crosstab

% within Bent_Needle

		Weekly_Dedication				
		<10 hours	11-20 hours	21-30 hours	31-40 hours	>40 hours
Bent_Needle	Never	7.7%	8.5%	18.5%	52.3%	13.1%
	Rarely	6.2%	9.3%	21.1%	40.4%	23.0%
	Sometimes	2.7%	9.7%	25.7%	41.6%	20.4%
	Often		18.8%	12.5%	50.0%	18.8%
Total		5.5%	9.5%	21.2%	44.8%	19.0%

Crosstab

% within Bent_Needle

		Total
Bent_Needle	Never	100.0%
	Rarely	100.0%
	Sometimes	100.0%
	Often	100.0%
Total		100.0%

Chi-Square Tests

	Value	df	Asymptotic Significance (2-sided)
Pearson Chi-Square	14.076[a]	12	.296
Likelihood Ratio	15.104	12	.236
Linear-by-Linear Association	.893	1	.345
N of Valid Cases	420		

a. 4 cells (20.0%) have expected count less than 5. The minimum expected count is .88.

Stuck_Needle * Hours_of_Training

Crosstab

% within Stuck_Needle

		Hours_of_Training				
		0-20 hours	20-60 hours	80-100 hours	>100 hours	Total
Stuck_Needle	Never	23.7%	36.9%	14.6%	24.7%	100.0%
	Rarely	15.5%	34.8%	22.6%	27.1%	100.0%
	Sometimes	22.4%	53.7%	3.0%	20.9%	100.0%
Total		20.5%	38.8%	15.7%	25.0%	100.0%

Chi-Square Tests

	Value	df	Asymptotic Significance (2-sided)
Pearson Chi-Square	20.132[a]	6	.003
Likelihood Ratio	22.832	6	<.001
Linear-by-Linear Association	.127	1	.722
N of Valid Cases	420		

a. 0 cells (0.0%) have expected count less than 5. The minimum expected count is 10.53.

Stuck_Needle * Professional_Experience

Crosstab

% within Stuck_Needle

		Professional_Experience				
		<2 years	3-5 years	6-10 years	>10 years	Total
Stuck_Needle	Never	43.4%	31.8%	21.2%	3.5%	100.0%
	Rarely	34.2%	30.3%	25.8%	9.7%	100.0%
	Sometimes	16.4%	44.8%	35.8%	3.0%	100.0%
Total		35.7%	33.3%	25.2%	5.7%	100.0%

Chi-Square Tests

	Value	df	Asymptotic Significance (2-sided)
Pearson Chi-Square	24.623[a]	6	<.001
Likelihood Ratio	25.375	6	<.001
Linear-by-Linear Association	12.364	1	<.001
N of Valid Cases	420		

a. 1 cells (8.3%) have expected count less than 5. The minimum expected count is 3.83.

Stuck_Needle * Percentage_of_DN_use

Crosstab

% within Stuck_Needle

		Percentage_of_DN_use					
		0-19%	20-39%	40-59%	60-79%	80-100%	Total
Stuck_Needle	Never	34.3%	38.4%	15.7%	10.1%	1.5%	100.0%
	Rarely	34.2%	23.2%	24.5%	16.8%	1.3%	100.0%
	Sometimes	17.2%	51.6%	18.8%	9.4%	3.1%	100.0%
Total		31.7%	34.8%	19.4%	12.5%	1.7%	100.0%

Chi-Square Tests

	Value	df	Asymptotic Significance (2-sided)
Pearson Chi-Square	25.058[a]	8	.002
Likelihood Ratio	25.658	8	.001
Linear-by-Linear Association	3.823	1	.051
N of Valid Cases	417		

a. 3 cells (20.0%) have expected count less than 5. The minimum expected count is 1.07.

Stuck_Needle * Weekly_Dedication

Crosstab

% within Stuck_Needle

		Weekly_Dedication				
		<10 hours	11-20 hours	21-30 hours	31-40 hours	>40 hours
Stuck_Needle	Never	2.5%	6.1%	19.2%	48.0%	24.2%
	Rarely	9.7%	10.3%	22.6%	40.6%	16.8%
	Sometimes	4.5%	17.9%	23.9%	44.8%	9.0%
Total		5.5%	9.5%	21.2%	44.8%	19.0%

Crosstab

% within Stuck_Needle

		Total
Stuck_Needle	Never	100.0%
	Rarely	100.0%
	Sometimes	100.0%
Total		100.0%

Chi-Square Tests

	Value	df	Asymptotic Significance (2-sided)
Pearson Chi-Square	24.409[a]	8	.002
Likelihood Ratio	24.303	8	.002
Linear-by-Linear Association	15.708	1	<.001
N of Valid Cases	420		

a. 1 cells (6.7%) have expected count less than 5. The minimum expected count is 3.67.

Broken_Needle * Hours_of_Training

Crosstab

% within Broken_Needle

		Hours_of_Training				Total
		0-20 hours	20-60 hours	80-100 hours	>100 hours	
Broken_Needle	Never	20.0%	38.8%	15.0%	26.3%	100.0%
	Rarely	30.0%	40.0%	30.0%		100.0%
Total		20.5%	38.8%	15.7%	25.0%	100.0%

Chi-Square Tests

	Value	df	Asymptotic Significance (2-sided)
Pearson Chi-Square	8.915[a]	3	.030
Likelihood Ratio	13.249	3	.004
Linear-by-Linear Association	3.705	1	.054
N of Valid Cases	420		

a. 2 cells (25.0%) have expected count less than 5. The minimum expected count is 3.14.

Broken_Needle * Professional_Experience

Crosstab

% within Broken_Needle

		Professional_Experience				Total
		<2 years	3-5 years	6-10 years	>10 years	
Broken_Needle	Never	33.5%	34.5%	26.0%	6.0%	100.0%
	Rarely	80.0%	10.0%	10.0%		100.0%
Total		35.7%	33.3%	25.2%	5.7%	100.0%

Chi-Square Tests

	Value	df	Asymptotic Significance (2-sided)
Pearson Chi-Square	18.094[a]	3	<.001
Likelihood Ratio	18.158	3	<.001
Linear-by-Linear Association	12.586	1	<.001
N of Valid Cases	420		

a. 1 cells (12.5%) have expected count less than 5. The minimum expected count is 1.14.

Broken_Needle * Percentage_of_DN_use

Crosstab

% within Broken_Needle

		Percentage_of_DN_use					
		0-19%	20-39%	40-59%	60-79%	80-100%	Total
Broken_Needle	Never	30.5%	36.5%	19.1%	12.1%	1.8%	100.0%
	Rarely	55.0%		25.0%	20.0%		100.0%
Total		31.7%	34.8%	19.4%	12.5%	1.7%	100.0%

Chi-Square Tests

	Value	df	Asymptotic Significance (2-sided)
Pearson Chi-Square	12.566[a]	4	.014
Likelihood Ratio	19.056	4	<.001
Linear-by-Linear Association	.111	1	.739
N of Valid Cases	417		

a. 3 cells (30.0%) have expected count less than 5. The minimum expected count is .34.

Broken_Needle * Weekly_Dedication

Crosstab

% within Broken_Needle

		Weekly_Dedication				
		<10 hours	11-20 hours	21-30 hours	31-40 hours	>40 hours
Broken_Needle	Never	4.0%	9.3%	21.0%	46.5%	19.3%
	Rarely	35.0%	15.0%	25.0%	10.0%	15.0%
Total		5.5%	9.5%	21.2%	44.8%	19.0%

Crosstab

% within Broken_Needle

		Total
Broken_Needle	Never	100.0%
	Rarely	100.0%
Total		100.0%

Chi-Square Tests

	Value	df	Asymptotic Significance (2-sided)
Pearson Chi-Square	40.081[a]	4	<.001
Likelihood Ratio	24.991	4	<.001
Linear-by-Linear Association	21.290	1	<.001
N of Valid Cases	420		

a. 4 cells (40.0%) have expected count less than 5. The minimum expected count is 1.10.

Lost_Needle * Hours_of_Training

Crosstab

% within Lost_Needle

		Hours_of_Training				Total
		0-20 hours	20-60 hours	80-100 hours	>100 hours	
Lost_Needle	Never	19.2%	39.9%	17.7%	23.2%	100.0%
	Rarely	20.3%	45.3%	8.1%	26.4%	100.0%
	Sometimes	22.4%	25.4%	25.4%	26.9%	100.0%
	Often	42.9%		28.6%	28.6%	100.0%
Total		20.5%	38.8%	15.7%	25.0%	100.0%

Chi-Square Tests

	Value	df	Asymptotic Significance (2-sided)
Pearson Chi-Square	20.613[a]	9	.014
Likelihood Ratio	23.663	9	.005
Linear-by-Linear Association	.208	1	.648
N of Valid Cases	420		

a. 4 cells (25.0%) have expected count less than 5. The minimum expected count is 1.10.

Lost_Needle * Professional_Experience

Crosstab

% within Lost_Needle

		Professional_Experience				Total
		<2 years	3-5 years	6-10 years	>10 years	
Lost_Needle	Never	37.4%	34.3%	23.2%	5.1%	100.0%
	Rarely	37.8%	31.8%	24.3%	6.1%	100.0%
	Sometimes	25.4%	31.3%	35.8%	7.5%	100.0%
	Often	42.9%	57.1%			100.0%
Total		35.7%	33.3%	25.2%	5.7%	100.0%

Chi-Square Tests

	Value	df	Asymptotic Significance (2-sided)
Pearson Chi-Square	9.937[a]	9	.356
Likelihood Ratio	11.748	9	.228
Linear-by-Linear Association	1.850	1	.174
N of Valid Cases	420		

a. 5 cells (31.3%) have expected count less than 5. The minimum expected count is .40.

Lost_Needle * Percentage_of_DN_use

Crosstab

% within Lost_Needle

		Percentage_of_DN_use					Total
		0-19%	20-39%	40-59%	60-79%	80-100%	
Lost_Needle	Never	30.8%	36.4%	23.6%	9.2%		100.0%
	Rarely	29.7%	40.5%	16.9%	10.8%	2.0%	100.0%
	Sometimes	41.8%	13.4%	11.9%	26.9%	6.0%	100.0%
	Often		71.4%	28.6%			100.0%
Total		31.7%	34.8%	19.4%	12.5%	1.7%	100.0%

Chi-Square Tests

	Value	df	Asymptotic Significance (2-sided)
Pearson Chi-Square	46.945[a]	12	<.001
Likelihood Ratio	49.826	12	<.001
Linear-by-Linear Association	3.119	1	.077
N of Valid Cases	417		

a. 8 cells (40.0%) have expected count less than 5. The minimum expected count is .12.

Lost_Needle * Weekly_Dedication

Crosstab

% within Lost_Needle

		Weekly_Dedication				
		<10 hours	11-20 hours	21-30 hours	31-40 hours	>40 hours
Lost_Needle	Never	6.1%	7.1%	20.2%	42.9%	23.7%
	Rarely	4.7%	10.8%	23.0%	47.3%	14.2%
	Sometimes	6.0%	9.0%	22.4%	44.8%	17.9%
	Often		57.1%		42.9%	
Total		5.5%	9.5%	21.2%	44.8%	19.0%

Crosstab

% within Lost_Needle

		Total
Lost_Needle	Never	100.0%
	Rarely	100.0%
	Sometimes	100.0%
	Often	100.0%
Total		100.0%

Chi-Square Tests

	Value	df	Asymptotic Significance (2-sided)
Pearson Chi-Square	26.593[a]	12	.009
Likelihood Ratio	20.915	12	.052
Linear-by-Linear Association	3.042	1	.081
N of Valid Cases	420		

a. 6 cells (30.0%) have expected count less than 5. The minimum expected count is .38.

Self_Needling * Hours_of_Training

Crosstab

% within Self_Needling

		Hours_of_Training				
		0-20 hours	20-60 hours	80-100 hours	>100 hours	Total
Self_Needling	Never	26.6%	35.6%	17.6%	20.3%	100.0%
	Rarely	11.5%	42.3%	14.8%	31.3%	100.0%
	Sometimes	37.5%	43.8%		18.8%	100.0%
Total		20.5%	38.8%	15.7%	25.0%	100.0%

Chi-Square Tests

	Value	df	Asymptotic Significance (2-sided)
Pearson Chi-Square	22.901[a]	6	<.001
Likelihood Ratio	25.882	6	<.001
Linear-by-Linear Association	3.385	1	.066
N of Valid Cases	420		

a. 3 cells (25.0%) have expected count less than 5. The minimum expected count is 2.51.

Self_Needling * Professional_Experience

Crosstab

% within Self_Needling

		Professional_Experience				
		<2 years	3-5 years	6-10 years	>10 years	Total
Self_Needling	Never	40.1%	33.3%	22.5%	4.1%	100.0%
	Rarely	32.4%	30.2%	29.1%	8.2%	100.0%
	Sometimes	12.5%	68.8%	18.8%		100.0%
Total		35.7%	33.3%	25.2%	5.7%	100.0%

Chi-Square Tests

	Value	df	Asymptotic Significance (2-sided)
Pearson Chi-Square	16.731[a]	6	.010
Likelihood Ratio	16.896	6	.010
Linear-by-Linear Association	4.991	1	.025
N of Valid Cases	420		

a. 2 cells (16.7%) have expected count less than 5. The minimum expected count is .91.

Self_Needling * Percentage_of_DN_use

Crosstab

% within Self_Needling

		Percentage_of_DN_use					
		0-19%	20-39%	40-59%	60-79%	80-100%	Total
Self_Needling	Never	32.4%	44.3%	18.3%	4.1%	0.9%	100.0%
	Rarely	30.8%	21.4%	21.4%	23.6%	2.7%	100.0%
	Sometimes	31.3%	56.3%	12.5%			100.0%
Total		31.7%	34.8%	19.4%	12.5%	1.7%	100.0%

Chi-Square Tests

	Value	df	Asymptotic Significance (2-sided)
Pearson Chi-Square	52.866[a]	8	<.001
Likelihood Ratio	56.371	8	<.001
Linear-by-Linear Association	10.393	1	.001
N of Valid Cases	417		

a. 5 cells (33.3%) have expected count less than 5. The minimum expected count is .27.

Self_Needling * Weekly_Dedication

Crosstab

% within Self_Needling

		Weekly_Dedication				
		<10 hours	11-20 hours	21-30 hours	31-40 hours	>40 hours
Self_Needling	Never	5.4%	9.0%	18.5%	43.7%	23.4%
	Rarely	6.0%	8.2%	24.7%	47.3%	13.7%
	Sometimes		31.3%	18.8%	31.3%	18.8%
Total		5.5%	9.5%	21.2%	44.8%	19.0%

Crosstab

% within Self_Needling

		Total
Self_Needling	Never	100.0%
	Rarely	100.0%
	Sometimes	100.0%
Total		100.0%

Chi-Square Tests

	Value	df	Asymptotic Significance (2-sided)
Pearson Chi-Square	17.075[a]	8	.029
Likelihood Ratio	15.159	8	.056
Linear-by-Linear Association	3.248	1	.071
N of Valid Cases	420		

a. 4 cells (26.7%) have expected count less than 5. The minimum expected count is .88.

Pneumothorax * Hours_of_Training

Crosstab

% within Pneumothorax

		Hours_of_Training				
		0-20 hours	20-60 hours	80-100 hours	>100 hours	Total
Pneumothorax	Never	21.1%	40.0%	13.4%	25.5%	100.0%
	Rarely	18.8%	15.6%	40.6%	25.0%	100.0%
	Sometimes			100.0%		100.0%
Total		20.8%	37.9%	15.9%	25.4%	100.0%

Chi-Square Tests

	Value	df	Asymptotic Significance (2-sided)
Pearson Chi-Square	29.000[a]	6	<.001
Likelihood Ratio	23.384	6	<.001
Linear-by-Linear Association	2.537	1	.111
N of Valid Cases	414		

a. 4 cells (33.3%) have expected count less than 5. The minimum expected count is .32.

Pneumothorax * Professional_Experience

Crosstab

% within Pneumothorax

		Professional_Experience				
		<2 years	3-5 years	6-10 years	>10 years	Total
Pneumothorax	Never	34.2%	33.7%	26.3%	5.8%	100.0%
	Rarely	46.9%	28.1%	18.8%	6.3%	100.0%
	Sometimes	100.0%				100.0%
Total		35.5%	33.1%	25.6%	5.8%	100.0%

Chi-Square Tests

	Value	df	Asymptotic Significance (2-sided)
Pearson Chi-Square	5.930[a]	6	.431
Likelihood Ratio	6.406	6	.379
Linear-by-Linear Association	2.964	1	.085
N of Valid Cases	414		

a. 5 cells (41.7%) have expected count less than 5. The minimum expected count is .12.

Pneumothorax * Percentage_of_DN_use

Crosstab

% within Pneumothorax

		Percentage_of_DN_use					
		0-19%	20-39%	40-59%	60-79%	80-100%	Total
Pneumothorax	Never	31.0%	36.3%	18.0%	12.7%	1.9%	100.0%
	Rarely	21.9%	25.0%	40.6%	12.5%		100.0%
	Sometimes	100.0%					100.0%
Total		30.7%	35.3%	19.7%	12.7%	1.7%	100.0%

Chi-Square Tests

	Value	df	Asymptotic Significance (2-sided)
Pearson Chi-Square	14.663[a]	8	.066
Likelihood Ratio	13.942	8	.083
Linear-by-Linear Association	.216	1	.642
N of Valid Cases	411		

a. 7 cells (46.7%) have expected count less than 5. The minimum expected count is .03.

Pneumothorax * Weekly_Dedication

Crosstab

% within Pneumothorax

		Weekly_Dedication				
		<10 hours	11-20 hours	21-30 hours	31-40 hours	>40 hours
Pneumothorax	Never	5.0%	8.7%	21.8%	45.0%	19.5%
	Rarely	12.5%	21.9%	12.5%	34.4%	18.8%
	Sometimes			100.0%		
Total		5.6%	9.7%	21.5%	44.0%	19.3%

Crosstab

% within Pneumothorax

		Total
Pneumothorax	Never	100.0%
	Rarely	100.0%
	Sometimes	100.0%
Total		100.0%

Chi-Square Tests

	Value	df	Asymptotic Significance (2-sided)
Pearson Chi-Square	17.606[a]	8	.024
Likelihood Ratio	14.691	8	.065
Linear-by-Linear Association	4.789	1	.029
N of Valid Cases	414		

a. 7 cells (46.7%) have expected count less than 5. The minimum expected count is .11.

Hematoma * Hours_of_Training

Crosstab

% within Hematoma

		Hours_of_Training				
		0-20 hours	20-60 hours	80-100 hours	>100 hours	Total
Hematoma	Never	32.3%	43.5%		24.2%	100.0%
	Rarely	12.7%	43.9%	15.9%	27.5%	100.0%
	Sometimes	24.7%	31.6%	21.5%	22.2%	100.0%
	Often	23.1%	23.1%	30.8%	23.1%	100.0%
Total		20.4%	38.6%	16.1%	24.9%	100.0%

Chi-Square Tests

	Value	df	Asymptotic Significance (2-sided)
Pearson Chi-Square	31.435[a]	9	<.001
Likelihood Ratio	41.089	9	<.001
Linear-by-Linear Association	.665	1	.415
N of Valid Cases	422		

a. 3 cells (18.8%) have expected count less than 5. The minimum expected count is 2.09.

Hematoma * Professional_Experience

Crosstab

% within Hematoma

		Professional_Experience				
		<2 years	3-5 years	6-10 years	>10 years	Total
Hematoma	Never	41.9%	29.0%	21.0%	8.1%	100.0%
	Rarely	33.3%	32.3%	28.0%	6.3%	100.0%
	Sometimes	35.4%	34.8%	25.3%	4.4%	100.0%
	Often	53.8%	46.2%			100.0%
Total		36.0%	33.2%	25.1%	5.7%	100.0%

Chi-Square Tests

	Value	df	Asymptotic Significance (2-sided)
Pearson Chi-Square	9.573[a]	9	.386
Likelihood Ratio	13.306	9	.149
Linear-by-Linear Association	.926	1	.336
N of Valid Cases	422		

a. 5 cells (31.3%) have expected count less than 5. The minimum expected count is .74.

Hematoma * Percentage_of_DN_use

Crosstab

% within Hematoma

		Percentage_of_DN_use					
		0-19%	20-39%	40-59%	60-79%	80-100%	Total
Hematoma	Never	30.6%	46.8%	8.1%	14.5%		100.0%
	Rarely	30.1%	33.3%	19.4%	16.1%	1.1%	100.0%
	Sometimes	36.1%	32.3%	21.5%	7.0%	3.2%	100.0%
	Often		23.1%	46.2%	30.8%		100.0%
Total		31.5%	34.6%	19.3%	12.9%	1.7%	100.0%

Chi-Square Tests

	Value	df	Asymptotic Significance (2-sided)
Pearson Chi-Square	30.993[a]	12	.002
Likelihood Ratio	35.334	12	<.001
Linear-by-Linear Association	.865	1	.352
N of Valid Cases	419		

a. 8 cells (40.0%) have expected count less than 5. The minimum expected count is .22.

Hematoma * Weekly_Dedication

Crosstab

% within Hematoma

		Weekly_Dedication				
		<10 hours	11-20 hours	21-30 hours	31-40 hours	>40 hours
Hematoma	Never	9.7%	6.5%	21.0%	40.3%	22.6%
	Rarely	2.6%	6.9%	20.1%	51.3%	19.0%
	Sometimes	6.3%	14.6%	20.3%	39.9%	19.0%
	Often	30.8%		46.2%	23.1%	
Total		5.9%	9.5%	21.1%	44.5%	19.0%

Crosstab

% within Hematoma

		Total
Hematoma	Never	100.0%
	Rarely	100.0%
	Sometimes	100.0%
	Often	100.0%
Total		100.0%

Chi-Square Tests

	Value	df	Asymptotic Significance (2-sided)
Pearson Chi-Square	37.194[a]	12	<.001
Likelihood Ratio	33.559	12	<.001
Linear-by-Linear Association	6.274	1	.012
N of Valid Cases	422		

a. 5 cells (25.0%) have expected count less than 5. The minimum expected count is .77.

Excessive_Bleeding * Hours_of_Training

Crosstab

% within Excessive_Bleeding

		Hours_of_Training			
		0-20 hours	20-60 hours	80-100 hours	>100 hours
Excessive_Bleeding	Never	24.5%	43.6%	8.3%	23.7%
	Rarely	15.9%	32.4%	25.9%	25.9%
	Sometimes		42.9%		57.1%
Total		20.6%	39.0%	15.3%	25.1%

Crosstab

% within Excessive_Bleeding

		Total
Excessive_Bleeding	Never	100.0%
	Rarely	100.0%
	Sometimes	100.0%
Total		100.0%

Chi-Square Tests

	Value	df	Asymptotic Significance (2-sided)
Pearson Chi-Square	32.614[a]	6	<.001
Likelihood Ratio	33.897	6	<.001
Linear-by-Linear Association	10.738	1	.001
N of Valid Cases	418		

a. 4 cells (33.3%) have expected count less than 5. The minimum expected count is 1.07.

Excessive_Bleeding * Professional_Experience

Crosstab

% within Excessive_Bleeding

		Professional_Experience				
		<2 years	3-5 years	6-10 years	>10 years	Total
Excessive_Bleeding	Never	39.8%	31.1%	22.4%	6.6%	100.0%
	Rarely	30.0%	37.1%	29.4%	3.5%	100.0%
	Sometimes	42.9%	28.6%		28.6%	100.0%
Total		35.9%	33.5%	24.9%	5.7%	100.0%

Chi-Square Tests

	Value	df	Asymptotic Significance (2-sided)
Pearson Chi-Square	15.763[a]	6	.015
Likelihood Ratio	14.578	6	.024
Linear-by-Linear Association	1.496	1	.221
N of Valid Cases	418		

a. 4 cells (33.3%) have expected count less than 5. The minimum expected count is .40.

Excessive_Bleeding * Percentage_of_DN_use

Crosstab

% within Excessive_Bleeding

		Percentage_of_DN_use				
		0-19%	20-39%	40-59%	60-79%	80-100%
Excessive_Bleeding	Never	36.5%	35.3%	17.4%	10.0%	0.8%
	Rarely	25.1%	34.1%	21.0%	16.8%	3.0%
	Sometimes		42.9%	57.1%		
Total		31.3%	34.9%	19.5%	12.5%	1.7%

Crosstab

% within Excessive_Bleeding

		Total
Excessive_Bleeding	Never	100.0%
	Rarely	100.0%
	Sometimes	100.0%
Total		100.0%

Chi-Square Tests

	Value	df	Asymptotic Significance (2-sided)
Pearson Chi-Square	19.656[a]	8	.012
Likelihood Ratio	20.934	8	.007
Linear-by-Linear Association	11.504	1	<.001
N of Valid Cases	415		

a. 7 cells (46.7%) have expected count less than 5. The minimum expected count is .12.

Excessive_Bleeding * Weekly_Dedication

Crosstab

% within Excessive_Bleeding

		Weekly_Dedication			
		<10 hours	11-20 hours	21-30 hours	31-40 hours
Excessive_Bleeding	Never	5.8%	9.5%	24.5%	46.1%
	Rarely	5.3%	8.8%	14.7%	44.1%
	Sometimes		28.6%	42.9%	28.6%
Total		5.5%	9.6%	20.8%	45.0%

Crosstab

% within Excessive_Bleeding

		Weekly_Dedi...	
		>40 hours	Total
Excessive_Bleeding	Never	14.1%	100.0%
	Rarely	27.1%	100.0%
	Sometimes		100.0%
Total		19.1%	100.0%

Chi-Square Tests

	Value	df	Asymptotic Significance (2-sided)
Pearson Chi-Square	20.027[a]	8	.010
Likelihood Ratio	20.453	8	.009
Linear-by-Linear Association	2.611	1	.106
N of Valid Cases	418		

a. 5 cells (33.3%) have expected count less than 5. The minimum expected count is .39.

Accidental_Nerve_Puncture * Hours_of_Training

Crosstab

% within Accidental_Nerve_Puncture

		Hours_of_Training			
		0-20 hours	20-60 hours	80-100 hours	>100 hours
Accidental_Nerve_Puncture	Never	22.4%	45.9%	9.2%	22.4%
	Rarely	16.1%	36.0%	20.5%	27.3%
	Sometimes	26.7%	25.0%	25.0%	23.3%
Total		20.6%	39.1%	15.8%	24.5%

Crosstab

% within Accidental_Nerve_Puncture

		Total
Accidental_Nerve_Puncture	Never	100.0%
	Rarely	100.0%
	Sometimes	100.0%
Total		100.0%

Chi-Square Tests

	Value	df	Asymptotic Significance (2-sided)
Pearson Chi-Square	20.485[a]	6	.002
Likelihood Ratio	21.147	6	.002
Linear-by-Linear Association	2.568	1	.109
N of Valid Cases	417		

a. 0 cells (0.0%) have expected count less than 5. The minimum expected count is 9.50.

Accidental_Nerve_Puncture * Professional_Experience

Crosstab

% within Accidental_Nerve_Puncture

		Professional_Experience			
		<2 years	3-5 years	6-10 years	>10 years
Accidental_Nerve_Puncture	Never	29.1%	36.7%	29.6%	4.6%
	Rarely	46.0%	27.3%	19.3%	7.5%
	Sometimes	31.7%	40.0%	23.3%	5.0%
Total		36.0%	33.6%	24.7%	5.8%

Crosstab

% within Accidental_Nerve_Puncture

		Total
Accidental_Nerve_Puncture	Never	100.0%
	Rarely	100.0%
	Sometimes	100.0%
Total		100.0%

Chi-Square Tests

	Value	df	Asymptotic Significance (2-sided)
Pearson Chi-Square	15.759[a]	6	.015
Likelihood Ratio	15.673	6	.016
Linear-by-Linear Association	1.759	1	.185
N of Valid Cases	417		

a. 1 cells (8.3%) have expected count less than 5. The minimum expected count is 3.45.

Accidental_Nerve_Puncture * Percentage_of_DN_use

Crosstab

% within Accidental_Nerve_Puncture

		Percentage_of_DN_use				
		0-19%	20-39%	40-59%	60-79%	80-100%
Accidental_Nerve_Puncture	Never	36.3%	37.8%	14.0%	11.9%	
	Rarely	30.4%	29.8%	21.7%	13.7%	4.3%
	Sometimes	21.7%	35.0%	31.7%	11.7%	
Total		31.9%	34.3%	19.6%	12.6%	1.7%

Crosstab

% within Accidental_Nerve_Puncture

		Total
Accidental_Nerve_Puncture	Never	100.0%
	Rarely	100.0%
	Sometimes	100.0%
Total		100.0%

Chi-Square Tests

	Value	df	Asymptotic Significance (2-sided)
Pearson Chi-Square	24.091[a]	8	.002
Likelihood Ratio	26.149	8	<.001
Linear-by-Linear Association	6.936	1	.008
N of Valid Cases	414		

a. 3 cells (20.0%) have expected count less than 5. The minimum expected count is 1.01.

Accidental_Nerve_Puncture * Weekly_Dedication

Crosstab

% within Accidental_Nerve_Puncture

		Weekly_Dedication			
		<10 hours	11-20 hours	21-30 hours	31-40 hours
Accidental_Nerve_Puncture	Never	7.1%	5.1%	20.9%	43.9%
	Rarely	3.1%	13.0%	24.8%	43.5%
	Sometimes	6.7%	15.0%	13.3%	48.3%
Total		5.5%	9.6%	21.3%	44.4%

Crosstab

% within Accidental_Nerve_Puncture

		Weekly_Dedi...	
		>40 hours	Total
Accidental_Nerve_Puncture	Never	23.0%	100.0%
	Rarely	15.5%	100.0%
	Sometimes	16.7%	100.0%
Total		19.2%	100.0%

Chi-Square Tests

	Value	df	Asymptotic Significance (2-sided)
Pearson Chi-Square	16.502[a]	8	.036
Likelihood Ratio	17.343	8	.027
Linear-by-Linear Association	1.914	1	.167
N of Valid Cases	417		

a. 1 cells (6.7%) have expected count less than 5. The minimum expected count is 3.31.

Accidental_Visceral_Puncture * Hours_of_Training

Crosstab

% within Accidental_Visceral_Puncture

		Hours_of_Training			
		0-20 hours	20-60 hours	80-100 hours	>100 hours
Accidental_Visceral_Puncture	Never	20.7%	40.6%	13.7%	25.1%
	Rarely	18.2%	18.2%	39.4%	24.2%
Total		20.5%	38.8%	15.7%	25.0%

Crosstab

% within Accidental_Visceral_Puncture

		Total
Accidental_Visceral_Puncture	Never	100.0%
	Rarely	100.0%
Total		100.0%

Chi-Square Tests

	Value	df	Asymptotic Significance (2-sided)
Pearson Chi-Square	16.806[a]	3	<.001
Likelihood Ratio	14.240	3	.003
Linear-by-Linear Association	1.847	1	.174
N of Valid Cases	420		

a. 0 cells (0.0%) have expected count less than 5. The minimum expected count is 5.19.

Accidental_Visceral_Puncture * Professional_Experience

Crosstab

% within Accidental_Visceral_Puncture

		Professional_Experience				
		<2 years	3-5 years	6-10 years	>10 years	Total
Accidental_Visceral_Puncture	Never	33.9%	34.6%	25.3%	6.2%	100.0%
	Rarely	57.6%	18.2%	24.2%		100.0%
Total		35.7%	33.3%	25.2%	5.7%	100.0%

Chi-Square Tests

	Value	df	Asymptotic Significance (2-sided)
Pearson Chi-Square	9.320[a]	3	.025
Likelihood Ratio	10.961	3	.012
Linear-by-Linear Association	5.012	1	.025
N of Valid Cases	420		

a. 1 cells (12.5%) have expected count less than 5. The minimum expected count is 1.89.

Accidental_Visceral_Puncture * Percentage_of_DN_use

Crosstab

% within Accidental_Visceral_Puncture

		Percentage_of_DN_use				
		0-19%	20-39%	40-59%	60-79%	80-100%
Accidental_Visceral_Puncture	Never	31.3%	36.7%	18.8%	11.5%	1.8%
	Rarely	36.4%	12.1%	27.3%	24.2%	
Total		31.7%	34.8%	19.4%	12.5%	1.7%

Crosstab

% within Accidental_Visceral_Puncture

		Total
Accidental_Visceral_Puncture	Never	100.0%
	Rarely	100.0%
Total		100.0%

Chi-Square Tests

	Value	df	Asymptotic Significance (2-sided)
Pearson Chi-Square	11.259[a]	4	.024
Likelihood Ratio	12.534	4	.014
Linear-by-Linear Association	1.484	1	.223
N of Valid Cases	417		

a. 2 cells (20.0%) have expected count less than 5. The minimum expected count is .55.

Accidental_Visceral_Puncture * Weekly_Dedication

Crosstab

% within Accidental_Visceral_Puncture

		Weekly_Dedication			
		<10 hours	11-20 hours	21-30 hours	31-40 hours
Accidental_Visceral_Puncture	Never	4.4%	8.5%	22.0%	45.7%
	Rarely	18.2%	21.2%	12.1%	33.3%
Total		5.5%	9.5%	21.2%	44.8%

Crosstab

% within Accidental_Visceral_Puncture

		Weekly_Dedi...	
		>40 hours	Total
Accidental_Visceral_Puncture	Never	19.4%	100.0%
	Rarely	15.2%	100.0%
Total		19.0%	100.0%

Chi-Square Tests

	Value	df	Asymptotic Significance (2-sided)
Pearson Chi-Square	18.416[a]	4	.001
Likelihood Ratio	13.889	4	.008
Linear-by-Linear Association	9.988	1	.002
N of Valid Cases	420		

a. 2 cells (20.0%) have expected count less than 5. The minimum expected count is 1.81.

Infection * Hours_of_Training

Crosstab

% within Infection

		Hours_of_Training				
		0-20 hours	20-60 hours	80-100 hours	>100 hours	Total
Infection	Never	19.7%	39.6%	15.2%	25.5%	100.0%
	Rarely	30.0%	20.0%	30.0%	20.0%	100.0%
Total		20.2%	38.7%	15.9%	25.2%	100.0%

Chi-Square Tests

	Value	df	Asymptotic Significance (2-sided)
Pearson Chi-Square	5.774[a]	3	.123
Likelihood Ratio	5.532	3	.137
Linear-by-Linear Association	.069	1	.793
N of Valid Cases	416		

a. 2 cells (25.0%) have expected count less than 5. The minimum expected count is 3.17.

Infection * Professional_Experience

Crosstab

% within Infection

		Professional_Experience				
		<2 years	3-5 years	6-10 years	>10 years	Total
Infection	Never	34.1%	34.1%	25.8%	6.1%	100.0%
	Rarely	55.0%	25.0%	20.0%		100.0%
Total		35.1%	33.7%	25.5%	5.8%	100.0%

Chi-Square Tests

	Value	df	Asymptotic Significance (2-sided)
Pearson Chi-Square	4.299[a]	3	.231
Likelihood Ratio	5.180	3	.159
Linear-by-Linear Association	3.417	1	.065
N of Valid Cases	416		

a. 1 cells (12.5%) have expected count less than 5. The minimum expected count is 1.15.

Infection * Percentage_of_DN_use

Crosstab

% within Infection

		Percentage_of_DN_use					
		0-19%	20-39%	40-59%	60-79%	80-100%	Total
Infection	Never	31.8%	35.6%	19.6%	11.7%	1.3%	100.0%
	Rarely	35.0%	15.0%	10.0%	30.0%	10.0%	100.0%
Total		32.0%	34.6%	19.1%	12.6%	1.7%	100.0%

Chi-Square Tests

	Value	df	Asymptotic Significance (2-sided)
Pearson Chi-Square	16.927[a]	4	.002
Likelihood Ratio	12.040	4	.017
Linear-by-Linear Association	4.174	1	.041
N of Valid Cases	413		

a. 3 cells (30.0%) have expected count less than 5. The minimum expected count is .34.

Infection * Weekly_Dedication

Crosstab

% within Infection

		Weekly_Dedication				
		<10 hours	11-20 hours	21-30 hours	31-40 hours	>40 hours
Infection	Never	4.3%	7.3%	21.5%	47.5%	19.4%
	Rarely	20.0%	45.0%	20.0%		15.0%
Total		5.0%	9.1%	21.4%	45.2%	19.2%

Crosstab

% within Infection

		Total
Infection	Never	100.0%
	Rarely	100.0%
Total		100.0%

Chi-Square Tests

	Value	df	Asymptotic Significance (2-sided)
Pearson Chi-Square	48.600[a]	4	<.001
Likelihood Ratio	40.144	4	<.001
Linear-by-Linear Association	27.190	1	<.001
N of Valid Cases	416		

a. 4 cells (40.0%) have expected count less than 5. The minimum expected count is 1.01.

Fainting * Hours_of_Training

Crosstab

% within Fainting

		Hours_of_Training				Total
		0-20 hours	20-60 hours	80-100 hours	>100 hours	
Fainting	Never	23.2%	35.0%	15.3%	26.6%	100.0%
	Rarely	18.5%	42.9%	16.4%	22.2%	100.0%
	Sometimes	14.3%	39.3%	14.3%	32.1%	100.0%
Total		20.5%	38.8%	15.7%	25.0%	100.0%

Chi-Square Tests

	Value	df	Asymptotic Significance (2-sided)
Pearson Chi-Square	4.638[a]	6	.591
Likelihood Ratio	4.655	6	.589
Linear-by-Linear Association	.146	1	.703
N of Valid Cases	420		

a. 1 cells (8.3%) have expected count less than 5. The minimum expected count is 4.40.

Fainting * Professional_Experience

Crosstab

% within Fainting

		Professional_Experience				Total
		<2 years	3-5 years	6-10 years	>10 years	
Fainting	Never	37.9%	37.9%	22.2%	2.0%	100.0%
	Rarely	31.2%	30.7%	27.5%	10.6%	100.0%
	Sometimes	50.0%	17.9%	32.1%		100.0%
Total		35.7%	33.3%	25.2%	5.7%	100.0%

Chi-Square Tests

	Value	df	Asymptotic Significance (2-sided)
Pearson Chi-Square	22.737[a]	6	<.001
Likelihood Ratio	24.662	6	<.001
Linear-by-Linear Association	3.216	1	.073
N of Valid Cases	420		

a. 1 cells (8.3%) have expected count less than 5. The minimum expected count is 1.60.

Fainting * Percentage_of_DN_use

Crosstab

% within Fainting

		Percentage_of_DN_use					
		0-19%	20-39%	40-59%	60-79%	80-100%	Total
Fainting	Never	36.5%	35.0%	14.5%	14.0%		100.0%
	Rarely	27.5%	34.4%	26.5%	7.9%	3.7%	100.0%
	Sometimes	25.0%	35.7%	7.1%	32.1%		100.0%
Total		31.7%	34.8%	19.4%	12.5%	1.7%	100.0%

Chi-Square Tests

	Value	df	Asymptotic Significance (2-sided)
Pearson Chi-Square	33.023[a]	8	<.001
Likelihood Ratio	33.904	8	<.001
Linear-by-Linear Association	5.590	1	.018
N of Valid Cases	417		

a. 4 cells (26.7%) have expected count less than 5. The minimum expected count is .47.

Fainting * Weekly_Dedication

Crosstab

% within Fainting

		Weekly_Dedication				
		<10 hours	11-20 hours	21-30 hours	31-40 hours	>40 hours
Fainting	Never	5.4%	8.4%	22.2%	43.8%	20.2%
	Rarely	2.6%	10.1%	20.6%	47.6%	19.0%
	Sometimes	25.0%	14.3%	17.9%	32.1%	10.7%
Total		5.5%	9.5%	21.2%	44.8%	19.0%

Crosstab

% within Fainting

		Total
Fainting	Never	100.0%
	Rarely	100.0%
	Sometimes	100.0%
Total		100.0%

Chi-Square Tests

	Value	df	Asymptotic Significance (2-sided)
Pearson Chi-Square	26.067[a]	8	.001
Likelihood Ratio	17.784	8	.023
Linear-by-Linear Association	3.712	1	.054
N of Valid Cases	420		

a. 2 cells (13.3%) have expected count less than 5. The minimum expected count is 1.53.

Allergy * Hours_of_Training

Crosstab

% within Allergy

		Hours_of_Training				Total
		0-20 hours	20-60 hours	80-100 hours	>100 hours	
Allergy	Never	18.2%	39.2%	16.8%	25.8%	100.0%
	Rarely	36.8%	35.1%	10.5%	17.5%	100.0%
Total		20.8%	38.6%	15.9%	24.6%	100.0%

Chi-Square Tests

	Value	df	Asymptotic Significance (2-sided)
Pearson Chi-Square	11.000[a]	3	.012
Likelihood Ratio	9.986	3	.019
Linear-by-Linear Association	7.264	1	.007
N of Valid Cases	414		

a. 0 cells (0.0%) have expected count less than 5. The minimum expected count is 9.09.

Allergy * Professional_Experience

Crosstab

% within Allergy

		Professional_Experience				Total
		<2 years	3-5 years	6-10 years	>10 years	
Allergy	Never	33.1%	36.1%	26.1%	4.8%	100.0%
	Rarely	56.1%	19.3%	17.5%	7.0%	100.0%
Total		36.2%	33.8%	24.9%	5.1%	100.0%

Chi-Square Tests

	Value	df	Asymptotic Significance (2-sided)
Pearson Chi-Square	13.274[a]	3	.004
Likelihood Ratio	13.140	3	.004
Linear-by-Linear Association	4.419	1	.036
N of Valid Cases	414		

a. 1 cells (12.5%) have expected count less than 5. The minimum expected count is 2.89.

Allergy * Percentage_of_DN_use

Crosstab

% within Allergy

		Percentage_of_DN_use					Total
		0-19%	20-39%	40-59%	60-79%	80-100%	
Allergy	Never	33.9%	32.8%	19.8%	11.6%	2.0%	100.0%
	Rarely	21.1%	45.6%	19.3%	14.0%		100.0%
Total		32.1%	34.5%	19.7%	11.9%	1.7%	100.0%

Chi-Square Tests

	Value	df	Asymptotic Significance (2-sided)
Pearson Chi-Square	6.248[a]	4	.181
Likelihood Ratio	7.317	4	.120
Linear-by-Linear Association	.562	1	.453
N of Valid Cases	411		

a. 1 cells (10.0%) have expected count less than 5. The minimum expected count is .97.

Allergy * Weekly_Dedication

Crosstab

% within Allergy

		Weekly_Dedication					Total
		<10 hours	11-20 hours	21-30 hours	31-40 hours	>40 hours	
Allergy	Never	4.8%	7.3%	21.0%	45.4%	21.6%	100.0%
	Rarely	10.5%	24.6%	24.6%	35.1%	5.3%	100.0%
Total		5.6%	9.7%	21.5%	44.0%	19.3%	100.0%

Chi-Square Tests

	Value	df	Asymptotic Significance (2-sided)
Pearson Chi-Square	26.363[a]	4	<.001
Likelihood Ratio	24.513	4	<.001
Linear-by-Linear Association	21.943	1	<.001
N of Valid Cases	414		

a. 1 cells (10.0%) have expected count less than 5. The minimum expected count is 3.17.

Myoedema * Hours_of_Training

Crosstab

% within Myoedema

		Hours_of_Training				
		0-20 hours	20-60 hours	80-100 hours	>100 hours	Total
Myoedema	Never	23.3%	37.8%	12.2%	26.7%	100.0%
	Rarely	16.0%	38.8%	14.4%	30.9%	100.0%
	Sometimes	23.6%	39.0%	21.1%	16.3%	100.0%
	Often	35.3%	35.3%	11.8%	17.6%	100.0%
Total		20.6%	38.5%	15.8%	25.1%	100.0%

Chi-Square Tests

	Value	df	Asymptotic Significance (2-sided)
Pearson Chi-Square	14.818[a]	9	.096
Likelihood Ratio	14.925	9	.093
Linear-by-Linear Association	2.395	1	.122
N of Valid Cases	418		

a. 3 cells (18.8%) have expected count less than 5. The minimum expected count is 2.68.

Myoedema * Professional_Experience

Crosstab

% within Myoedema

		Professional_Experience				
		<2 years	3-5 years	6-10 years	>10 years	Total
Myoedema	Never	62.2%	20.0%	8.9%	8.9%	100.0%
	Rarely	30.3%	38.3%	26.6%	4.8%	100.0%
	Sometimes	25.2%	35.8%	33.3%	5.7%	100.0%
	Often	35.3%	35.3%	29.4%		100.0%
Total		35.9%	33.5%	24.9%	5.7%	100.0%

Chi-Square Tests

	Value	df	Asymptotic Significance (2-sided)
Pearson Chi-Square	45.301[a]	9	<.001
Likelihood Ratio	46.941	9	<.001
Linear-by-Linear Association	12.703	1	<.001
N of Valid Cases	418		

a. 2 cells (12.5%) have expected count less than 5. The minimum expected count is .98.

Myoedema * Percentage_of_DN_use

Crosstab

% within Myoedema

		Percentage_of_DN_use					Total
		0-19%	20-39%	40-59%	60-79%	80-100%	
Myoedema	Never	31.1%	51.1%	12.2%	5.6%		100.0%
	Rarely	33.5%	29.7%	20.0%	15.7%	1.1%	100.0%
	Sometimes	34.1%	26.8%	22.8%	12.2%	4.1%	100.0%
	Often		52.9%	29.4%	17.6%		100.0%
Total		31.8%	34.5%	19.5%	12.5%	1.7%	100.0%

Chi-Square Tests

	Value	df	Asymptotic Significance (2-sided)
Pearson Chi-Square	33.654[a]	12	<.001
Likelihood Ratio	39.792	12	<.001
Linear-by-Linear Association	7.515	1	.006
N of Valid Cases	415		

a. 6 cells (30.0%) have expected count less than 5. The minimum expected count is .29.

Myoedema * Weekly_Dedication

Crosstab

% within Myoedema

		Weekly_Dedication				
		<10 hours	11-20 hours	21-30 hours	31-40 hours	>40 hours
Myoedema	Never	8.9%	5.6%	21.1%	43.3%	21.1%
	Rarely	3.2%	9.0%	25.5%	36.2%	26.1%
	Sometimes	7.3%	12.2%	13.0%	57.7%	9.8%
	Often		17.6%	35.3%	47.1%	
Total		5.5%	9.6%	21.3%	44.5%	19.1%

Crosstab

% within Myoedema

		Total
Myoedema	Never	100.0%
	Rarely	100.0%
	Sometimes	100.0%
	Often	100.0%
Total		100.0%

Chi-Square Tests

	Value	df	Asymptotic Significance (2-sided)
Pearson Chi-Square	37.728[a]	12	<.001
Likelihood Ratio	42.680	12	<.001
Linear-by-Linear Association	2.136	1	.144
N of Valid Cases	418		

a. 5 cells (25.0%) have expected count less than 5. The minimum expected count is .94.
